# Pembrolizumab and chemotherapy in high-risk, early-stage, ER^+^/HER2^−^ breast cancer: a randomized phase 3 trial

**DOI:** 10.1038/s41591-024-03415-7

**Published:** 2025-01-21

**Authors:** Fatima Cardoso, Joyce O’Shaughnessy, Zhenzhen Liu, Heather McArthur, Peter Schmid, Javier Cortes, Nadia Harbeck, Melinda L. Telli, David W. Cescon, Peter A. Fasching, Zhimin Shao, Delphine Loirat, Yeon Hee Park, Manuel Gonzalez Fernandez, Gábor Rubovszky, Laura Spring, Seock-Ah Im, Rina Hui, Toshimi Takano, Fabrice André, Hiroyuki Yasojima, Yu Ding, Liyi Jia, Vassiliki Karantza, Konstantinos Tryfonidis, Aditya Bardia

**Affiliations:** 1 Breast Unit, Champalimaud Clinical Centre/Champalimaud Foundation, Lisbon, Portugal; 2 Baylor University Medical Center, Texas Oncology, US Oncology Network, Dallas, TX USA; 3 Department of Breast Disease, Henan Breast Cancer Center, Affiliated Cancer Hospital of Zhengzhou University & Henan Cancer Hospital, Zhengzhou, China; 4 University of Texas Southwestern Medical Center, Dallas, TX USA; 5 Centre for Experimental Cancer Medicine, Barts Cancer Institute, Queen Mary University London, London, UK; 6 Medica Scientia Innovation Research, Ridgewood, NJ USA; 7 Medica Scientia Innovation Research, Barcelona, Spain; 8 International Breast Cancer Center (IBCC), Pangaea Oncology, Quiron Group, Barcelona, Spain; 9 Universidad Europea de Madrid Faculty of Biomedical and Health Sciences, Department of Medicine, Madrid, Spain; 10 IOB Madrid, Institute of Oncology, Hospital Beata María Ana, Madrid, Spain; 11 Breast Center, Department of OB&GYN, LMU University Hospital, Munich, Germany; 12 Stanford University School of Medicine, Stanford, CA USA; 13 Princess Margaret Cancer Centre, University Health Network, Toronto, Ontario Canada; 14 University Hospital Erlangen, Comprehensive Cancer Center Erlangen-EMN, Bavarian Cancer Research Center (BZKF), Erlangen, Germany; 15 Department of Breast Surgery, Fudan University Shanghai Cancer Center, Department of Oncology, Shanghai Medical College, Fudan University, Shanghai, China; 16 Institute Curie, Paris, France; 17 Samsung Medical Division, Sungkyunkwan University School of Medicine, Seoul, Republic of Korea; 18 Hemato Oncólogo, IMAT Oncomedica, Montería, Colombia; 19 National Institute of Oncology, Budapest, Hungary; 20 Massachusetts General Hospital and Harvard Medical School, Boston, MA USA; 21 Seoul National University Hospital, Cancer Research Institute, Seoul National University College of Medicine, Seoul National University, Seoul, Republic of Korea; 22 Westmead Breast Cancer Institute, Westmead Hospital and the University of Sydney, Sydney, New South Wales Australia; 23 Centre of Cancer Medicine, University of Hong Kong, Pokfulam, Hong Kong; 24 The Cancer Institute Hospital of JFCR, Tokyo, Japan; 25 Faculté de Médecine, Paris Saclay University, Gustave Roussy, Villejuif, France; 26 NHO Osaka National Hospital, Osaka, Japan; 27 Merck & Co., Inc., Rahway, NJ USA; 28 University of California Los Angeles, Jonsson Comprehensive Cancer Center, Los Angeles, CA USA

**Keywords:** Cancer therapy, Breast cancer

## Abstract

Addition of pembrolizumab to neoadjuvant chemotherapy followed by adjuvant pembrolizumab improved outcomes in patients with high-risk, early-stage, triple-negative breast cancer. However, whether the addition of neoadjuvant pembrolizumab to chemotherapy would improve outcomes in high-risk, early-stage, estrogen receptor-positive/human epidermal growth factor receptor 2-negative (ER^+^/HER2^−^) breast cancer remains unclear. We conducted a double-blind, placebo-controlled phase 3 study (KEYNOTE-756) in which patients with previously untreated ER^+^/HER2^−^ grade 3 high-risk invasive breast cancer (T1c-2 (≥2 cm), cN1–2 or T3–4, cN0–2) were randomly assigned (1:1) to neoadjuvant pembrolizumab 200 mg or placebo Q3W given with paclitaxel QW for 12 weeks, followed by four cycles of doxorubicin or epirubicin plus cyclophosphamide Q2W or Q3W. After surgery (with/without adjuvant radiation therapy), patients received adjuvant pembrolizumab or placebo for nine cycles plus adjuvant endocrine therapy. Dual primary endpoints were pathological complete response and event-free survival in the intention-to-treat population. In total, 635 patients were assigned to the pembrolizumab−chemotherapy arm and 643 to the placebo−chemotherapy arm. At the study’s prespecified first interim analysis, the pathological complete response rate was 24.3% (95% confidence interval (CI), 21.0–27.8%) in the pembrolizumab−chemotherapy arm and 15.6% (95% CI, 12.8–18.6%) in the placebo−chemotherapy arm (estimated treatment difference, 8.5 percentage points; 95% CI, 4.2–12.8; *P* = 0.00005). Event-free survival was not mature in this analysis. During the neoadjuvant phase, treatment-related adverse events of grade ≥3 were reported in 52.5% and 46.4% of patients in the pembrolizumab−chemotherapy and placebo−chemotherapy arms, respectively. In summary, the addition of pembrolizumab to neoadjuvant chemotherapy significantly improved the pathological complete response rate in patients with high-risk, early-stage ER^+^/HER2^−^ breast cancer. Safety was consistent with the known profiles of each study treatment. Follow-up continues for event-free survival. ClinicalTrials.gov identifier: NCT03725059.

## Abstract (web summary)

In this prespecified interim analysis of the KEYNOTE-756 phase 3 trial, pembrolizumab and chemotherapy treatment of patients with high-risk, early-stage, estrogen receptor-positive/human epidermal growth factor receptor-negative breast cancer improved the pathological complete response rate compared with chemotherapy alone.

## Main

Estrogen receptor-positive/human epidermal growth factor receptor 2-negative (ER^+^/HER2^−^) breast cancer is a heterogeneous disease that includes a subpopulation of patients (including those with high-grade tumors and lymph node involvement) at high risk of recurrence who have poor long-term outcomes despite (neo)adjuvant chemotherapy and adjuvant endocrine therapy^1^. For these patients, reported pathological complete response (pCR) rates range from 0% to 18%^2^ and event-free survival (EFS) rates are similar to those of patients with triple-negative breast cancer (TNBC)^3^. Although ER^+^ disease is heterogeneous, in patients with ER^+^/HER2^−^ breast cancer, a meta-analysis of neoadjuvant studies has demonstrated a positive correlation between pCR and both EFS and overall survival (OS)^3^. Regulatory guidance supports pCR as an appropriate endpoint for the evaluation of the efficacy of neoadjuvant treatment^4,5^.

Combination therapy with immune checkpoint inhibitors plus chemotherapy induces changes to the tumor microenvironment that may enhance endogenous anticancer immunity, reduce tumor volume and increase response rate compared with chemotherapy alone^6,7^. Supporting this hypothesis, clinical data have demonstrated that the antiprogrammed cell death protein 1 (anti-PD-1) monoclonal antibody pembrolizumab combined with neoadjuvant chemotherapy in the phase 2 I-SPY2 trial more than doubled the estimated pCR rates for patients with high-risk (defined by MammaPrint score) ER^+^/HER2^−^ tumors compared with patients receiving neoadjuvant chemotherapy alone (30% versus 13%, respectively)^8^. Additionally, pembrolizumab combined with neoadjuvant chemotherapy has been shown to improve pCR and EFS in patients with early-stage TNBC^9,10^.

Building on these results, KEYNOTE-756 (NCT03725059) was designed as a randomized, double-blind, phase 3 study to evaluate the efficacy and safety of neoadjuvant pembrolizumab plus chemotherapy followed by adjuvant pembrolizumab plus endocrine therapy versus neoadjuvant placebo plus chemotherapy followed by adjuvant placebo plus endocrine therapy in patients with high-risk, early-stage, ER^+^/HER2^−^ breast cancer.

## Results

### Patients and treatment

**Fig. 1:**
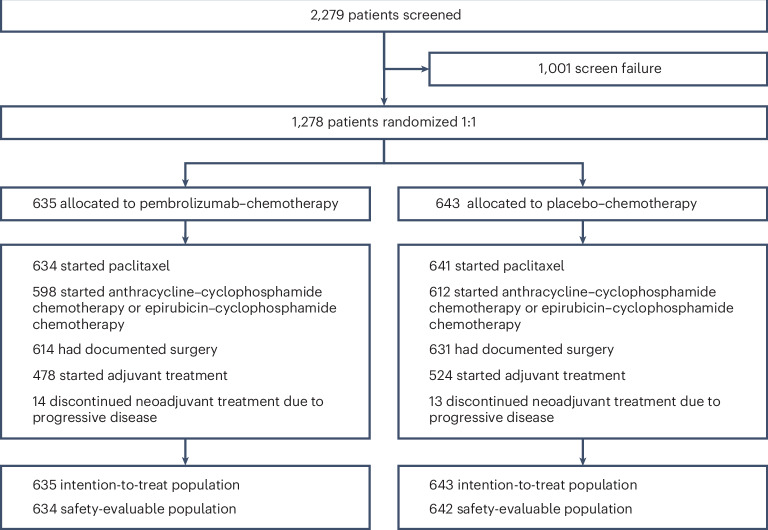
Disposition of patients in the study. Progressive disease included radiographic progressive disease. Patients did not have to complete all neoadjuvant therapy to undergo surgery.

From 27 December 2018 to 5 August 2022, a total of 1,278 patients from 222 global sites were randomly assigned to the pembrolizumab–chemotherapy arm (635 patients) or to the placebo–chemotherapy arm (643 patients; Fig. 1). Demographics and baseline disease characteristics were balanced between treatment arms (Table 1).

**Table 1:** Demographics and baseline disease characteristics^a^

Characteristic	Pembrolizumab–chemotherapy (*n* = 635)	Placebo–chemotherapy (*n* = 643)
Age (year)		
Median (range)	49 (24–82)	49 (19–78)
≥65—no. (%)	89 (14.0)	76 (11.8)
Country/region—no. (%)		
China	88 (13.9)	91 (14.2)
Eastern Europe	139 (21.9)	130 (20.2)
Other	408 (64.3)	422 (65.6)
PD-L1 CPS—no. (%)^b^		
<1	153 (24.1)	154 (24.0)
≥1	482 (75.9)	489 (76.0)
≥10	253 (39.8)	259 (40.3)
≥20	125 (19.7)	129 (20.1)
ECOG performance status—no. (%)^c^		
0	570 (89.8)	588 (91.4)
1	65 (10.2)	55 (8.6)
Anthracycline schedule—no. (%)		
Every 3 weeks	415 (65.4)	425 (66.1)
Every 2 weeks	183 (28.8)	187 (29.1)
Not started	37 (5.8)	31 (4.8)
Tumor classification—no. (%)		
T1–T2	402 (63.3)	413 (64.2)
T3–T4	233 (36.7)	230 (35.8)
Nodal involvement—no. (%)		
Positive	570 (89.8)	582 (90.5)
Negative	65 (10.2)	61 (9.5)
Overall disease stage—no. (%)		
Stage II	399 (62.8)	408 (63.5)
Stage III	236 (37.2)	235 (36.5)
Tumor grade—no. (%)		
Grade 3	635 (100)	642 (99.8)
Grade 2	0	1 (0.2)^d^
ER positivity—no. (%)		
≥10%	601 (94.6)	600 (93.3)
<10%	34 (5.4)	43 (6.7)
Menopausal status—no. (%)		
Premenopausal	354 (55.7)	353 (54.9)
Postmenopausal	278 (43.8)	287 (44.6)
Not applicable	3 (0.5)	3 (0.5)

Data are from the intention-to-treat population. All patients had previously untreated, centrally confirmed ER^+^, HER2^−^ disease.

^a^A total of six men with ER^+^ and HER2^−^ breast cancer were included in the study (three in each treatment arm).

^b^PD-L1 CPS was defined as the number of PD-L1-positive tumor cells, lymphocytes and macrophages divided by the total number of tumor cells multiplied by 100.

^c^ECOG performance status ranges from 0 to 5, with higher scores indicating greater disability.

^d^Protocol violation.

At the first interim analysis (data cutoff, 25 May 2023; median duration of follow-up, 33.2 (range = 9.7–51.8) months), 1,275 patients had received the first neoadjuvant treatment, 1,210 patients had started the second neoadjuvant treatment, 1,245 patients had documented surgery and 1,002 patients had started adjuvant treatment (Fig. 1). Median duration of treatment in the neoadjuvant phase was 4.9 months (range = 0.0–6.9 months) in the pembrolizumab–chemotherapy arm and 4.9 months (range = 0.0–7.8 months) in the placebo–chemotherapy arm (Extended Data Table 1). Both groups received a similar median number of chemotherapy cycles.

### Efficacy

A pCR (ypT0/Tis ypN0) was observed in 154 of 635 patients (24.3%) in the pembrolizumab–chemotherapy arm and 100 of 643 patients (15.6%) in the placebo‒chemotherapy arm. The estimated treatment difference in the rate of pCR was 8.5 percentage points (95% confidence interval (CI), 4.2–12.8; *P* = 0.00005; Table 2). The prespecified statistical significance criterion for this analysis was *P* = 0.005; thus, the percentage of patients who had a pCR was significantly higher in the pembrolizumab‒chemotherapy arm than in the placebo‒chemotherapy arm. Similar results were observed with respect to the percentage of patients who had a pCR defined per the secondary endpoints of ypT0 ypN0 and ypT0/Tis (Table 2).

**Table 2:** pCR at the first interim analysis according to pathological stage

Endpoint	Pembrolizumab–chemotherapy (*n* = 635)	Placebo–chemotherapy (*n* = 643)	Estimated treatment difference^a^ Percentage points (95% CI)	*P* value
Pathological stage ypT0/Tis ypN0				
Number of patients	154	100	–	–
Percentage of patients with response (95% CI)	24.3 (21.0–27.8)	15.6 (12.8–18.6)	8.5 (4.2–12.8)	0.00005
Pathological stage ypT0 ypN0				
Number of patients	135	82	–	–
Percentage of patients with response (95% CI)	21.3 (18.1–24.7)	12.8 (10.3–15.6)	8.3 (4.2–12.4)	–
Pathological stage ypT0/Tis				
Number of patients	187	117	–	–
Percentage of patients with response (95% CI)	29.4 (25.9–33.2)	18.2 (15.3–21.4)	11.0 (6.5–15.7)	–

Participants were considered non-responders if they did not receive the study medication, discontinued study treatment and continued neoadjuvant treatment with drug categories not specified by the study before surgery (regardless of surgical outcome), discontinued study treatment for reasons that precluded surgery or had missing data for pCR for any reason. pCR was assessed by the local pathologist at the time of surgery per the current AJCC breast cancer staging criteria. Pathological stage ypT0/Tis ypN0 was defined as the absence of residual invasive cancer in the complete resected breast specimen and all sampled regional lymph nodes. Pathological stage ypT0 ypN0 was defined as the absence of residual invasive and in situ cancer in the complete resected breast specimen and all sampled regional lymph nodes. Pathological stage ypT0/Tis was defined as the absence of residual invasive and in situ cancer in the complete resected breast specimen (independent of lymph node involvement) and all sampled regional lymph nodes.

^a^Estimated treatment difference was calculated using the stratified Miettinen–Nurminen method.

**Fig. 2:**
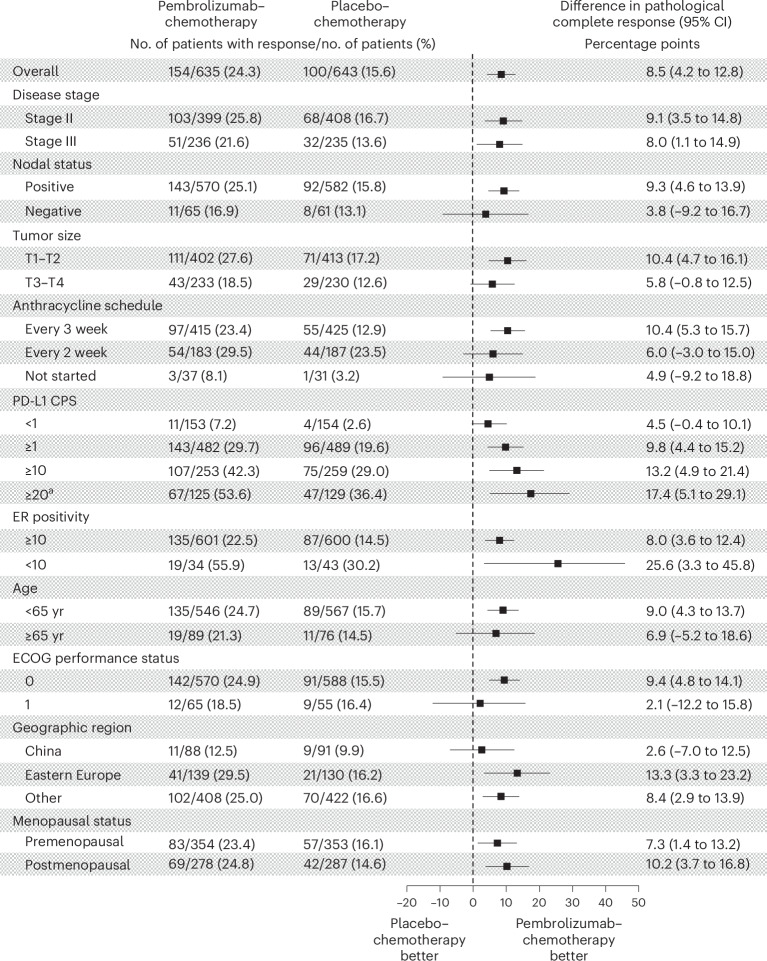
Subgroup analysis of the difference in percentages of patients with a pCR at the first interim analysis. Data from key subgroups are shown. For the overall population, the estimated mean treatment difference with 95% CI was calculated using the stratified Miettinen–Nurminen method. The analysis for PD-L1 CPS subgroups was stratified. All other analyses were unstratified. The footnote ‘a’ indicates the subgroup analyses of pCR based on a PD-L1 CPS cutoff of 20 were not prespecified. PD-L1 CPS was defined as the number of PD-L1-positive tumor cells, lymphocytes and macrophages divided by the total number of tumor cells multiplied by 100. ECOG performance status ranges from 0 to 5, with higher scores indicating greater disability.

Benefits of pembrolizumab‒chemotherapy as compared to placebo‒chemotherapy with respect to pCR (ypT0/Tis ypN0) were generally consistent across subgroups defined by demographics and baseline clinical characteristics (Fig. 2). Notably, a numerically higher rate of pCR difference was observed with higher tumor PD-L1 expression. The estimated treatment differences in the prespecified subgroups based on PD-L1 combined positive score (CPS) of <1 (*n* = 307), ≥1 (*n* = 971) and ≥10 (*n* = 512) were 4.5 (95% CI, −0.4 to 10.1), 9.8 (95% CI, 4.4–15.2) and 13.2 (95% CI, 4.9–21.4) percentage points, respectively. The estimated treatment difference was 17.4 (95% CI, 5.1–29.1) percentage points in the post hoc subgroup analysis of pCR based on PD-L1 CPS of ≥20 (*n* = 254). Additionally, pCR benefit for pembrolizumab‒chemotherapy was observed both in patients with ER positivity <10% and ≥10%, albeit with a greater magnitude among those with ER positivity <10%. Among patients with ER positivity <10%, the pCR rate was 55.9% (19 of 34 patients) in the pembrolizumab‒chemotherapy arm versus 30.2% (13 of 43 patients) in the placebo‒chemotherapy arm (estimated treatment difference, 25.6 percentage points (95% CI, 3.3–45.8%)), whereas among patients with ER positivity ≥10%, the pCR was 22.5% (135 of 601 patients) versus 14.5% (87 of 600 patients; estimated treatment difference, 8.0 percentage points (95% CI, 3.6–12.3%); Fig. 2).

**Extended Data Fig. 1:**
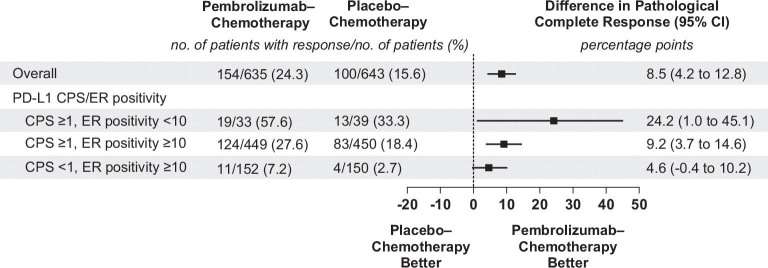
Post hoc exploratory analysis of the difference in percentages of patients with a pathological complete response by estrogen receptor positivity and PD-L1 combined positive score at the first interim analysis. Programmed cell death ligand 1 (PD-L1) combined positive score (CPS) was defined as the number of PD-L1-positive tumor cells, lymphocytes and macrophages divided by the total number of tumor cells multiplied by 100. No pathological complete response occurred in patients with tumor PD-L1 CPS < 1 with estrogen receptor (ER) positivity <10% (pembrolizumab arm, n = 1; placebo arm, n = 4). For the overall population, the estimated treatment difference was calculated using the Miettinen–Nurminen method stratified by region (China vs Eastern Europe vs all other countries) and PD-L1 status (CPS ≥ 1 vs <1). Estimated treatment differences for other endpoints were based on the Miettinen–Nurminen method (unstratified).

In post hoc exploratory analyses, an improvement in pCR (ypT0/Tis ypN0) was observed based on both PD-L1 CPSs and ER positivity, with the largest numerical difference observed in patients with PD-L1 CPS ≥1 and ER positivity <10% (*n* = 72; treatment difference, 24.2 percentage points (95% CI, 1.0–45.1); Extended Data Fig. 1).

**Fig. 3:**
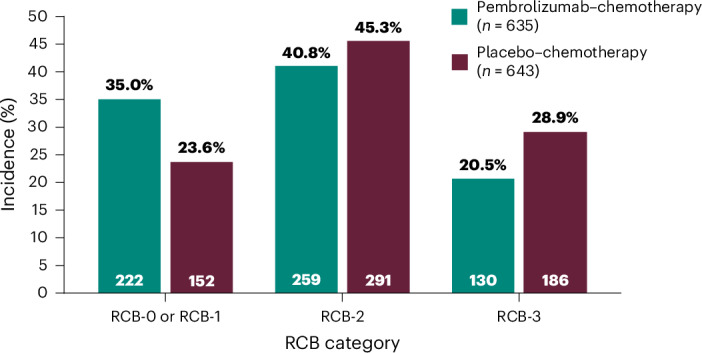
RCB at the first interim analysis. RCB was an exploratory endpoint and was assessed by a local pathologist at the time of surgery. RCB-0, RCB-1, RCB-2 and RCB-3 denote increasingly larger residual diseases. RCB data were missing for 24 (3.8%) and 14 patients (2.2%) in the pembrolizumab‒chemotherapy and placebo‒chemotherapy arms, respectively.

Analysis of the exploratory endpoint of residual cancer burden (RCB) showed that the addition of pembrolizumab to neoadjuvant chemotherapy shifted more patients to lower RCB categories (RCB-0 or RCB-1, 35.0% versus 23.6%; RCB-2, 40.8% versus 45.3%; RCB-3, 20.5% versus 28.9%; Fig. 3).

EFS was not mature at the first interim analysis; this endpoint continues to be evaluated by prespecified subsequent interim analyses and a final analysis.

### Safety

During the neoadjuvant phase, treatment-related adverse events (AEs) of any grade occurred in 624 of 634 patients (98.4%) in the pembrolizumab‒chemotherapy arm and 633 of 642 patients (98.6%) in the placebo‒chemotherapy arm (Table 3). Treatment-related grade ≥3 AEs occurred in 333 (52.5%) and 298 patients (46.4%), respectively. Serious treatment-related AEs were reported in 18.5% and 10.3% of patients, respectively. One patient (0.2%) in the pembrolizumab arm died due to treatment-related acute non-*Q* wave myocardial infarction. Discontinuation of any study treatment due to treatment-related AEs occurred in 121 patients (19.1%) in the pembrolizumab‒chemotherapy arm and 65 patients (10.1%) in the placebo‒chemotherapy arm. The most frequently occurring treatment-related AEs were alopecia (64.0% and 60.9%, respectively), nausea (48.3% and 50.0%, respectively), fatigue (30.0% and 28.0%, respectively) and anemia (32.3% and 25.5%, respectively; Table 3).

**Table 3:** AEs during the neoadjuvant phase at the first interim analysis

Adverse event	Pembrolizumab–chemotherapy (*n* = 634)		Placebo–chemotherapy (*n* = 642)	
	Any grade	Grade ≥3	Any grade	Grade ≥3
	Number of patients (%)			
Any AE	634 (100.0)	381 (60.1)	638 (99.4)	350 (54.5)
Treatment-related AE^a^	624 (98.4)	333 (52.5)	633 (98.6)	298 (46.4)
Alopecia	406 (64.0)	0	391 (60.9)	0
Nausea	306 (48.3)	8 (1.3)	321 (50.0)	7 (1.1)
Anemia	205 (32.3)	21 (3.3)	164 (25.5)	18 (2.8)
Fatigue	190 (30.0)	17 (2.7)	180 (28.0)	9 (1.4)
Diarrhea	172 (27.1)	11 (1.7)	130 (20.2)	10 (1.6)
Alanine aminotransferase increased	158 (24.9)	24 (3.8)	147 (22.9)	18 (2.8)
Neutropenia	146 (23.0)	85 (13.4)	158 (24.6)	101 (15.7)
Aspartate aminotransferase increased	137 (21.6)	10 (1.6)	107 (16.7)	6 (0.9)
Neutrophil count decreased	137 (21.6)	89 (14.0)	153 (23.8)	103 (16.0)
Asthenia	134 (21.1)	10 (1.6)	116 (18.1)	4 (0.6)
Vomiting	127 (20.0)	9 (1.4)	108 (16.8)	9 (1.4)
Peripheral neuropathy	111 (17.5)	4 (0.6)	130 (20.2)	5 (0.8)
Immune-mediated AE^b^	208 (32.8)	45 (7.1)	45 (7.0)	8 (1.2)
Hypothyroidism	111 (17.5)	1 (0.2)	11 (1.7)	0
Hyperthyroidism	57 (9.0)	1 (0.2)	3 (0.5)	0
Pneumonitis	18 (2.8)	9 (1.4)	9 (1.4)	2 (0.3)
Adrenal insufficiency	16 (2.5)	5 (0.8)	0	0
Severe skin reactions	14 (2.2)	8 (1.3)	3 (0.5)	2 (0.3)
Hypophysitis	12 (1.9)	6 (0.9)	1 (0.2)	0
Thyroiditis	11 (1.7)	1 (0.2)	2 (0.3)	0
Hepatitis	8 (1.3)	7 (1.1)	3 (0.5)	0
Colitis	6 (0.9)	3 (0.5)	5 (0.8)	1 (0.2)
Vasculitis	5 (0.8)	1 (0.2)	4 (0.6)	0

AEs were collected up to 30 days after discontinuation of treatment (90 days for serious AEs). Events are listed in descending order of frequency in the pembrolizumab‒chemotherapy arm. The safety-evaluable population included patients who received at least one trial drug, underwent surgery, or both. The severity of AEs was graded per the National Cancer Institute Common Terminology Criteria for AEs (v4.0).

^a^Treatment-related AEs were events that were considered related to a study treatment by the investigator. Treatment-related AEs that occurred in at least 20% of patients are reported.

^b^Immune-mediated AEs, excluding infusion reactions, were based on a list of preferred terms intended to capture known risks of pembrolizumab and were considered regardless of attribution to study treatment by the investigator. Immune-mediated AEs that occurred in ≥5 patients are reported.

Immune-mediated AEs, excluding infusion reactions, of any grade were observed in 208 patients (32.8%) in the pembrolizumab‒chemotherapy arm and 45 patients (7.0%) in the placebo‒chemotherapy arm (Table 3). Grade 3/4 immune-mediated AEs were reported in 45 (7.1%) and 8 patients (1.2%), respectively. The most frequently occurring immune-mediated AEs were hypothyroidism (17.5% and 1.7%, respectively), hyperthyroidism (9.0% and 0.5%, respectively) and pneumonitis (2.8% and 1.4%, respectively). No deaths were attributed to immune-mediated AEs.

## Discussion

In this randomized phase 3 trial involving patients with previously untreated, high-risk, early-stage, ER^+^/HER2^−^ breast cancer, a significantly higher percentage of patients in the pembrolizumab‒chemotherapy arm than in the placebo‒chemotherapy arm had a pCR at the time of surgery. The between-group difference in pCR favored pembrolizumab‒chemotherapy across all prespecified subgroups, albeit with a differing magnitude of benefit and wide 95% CIs in some subgroups. Notably, pCR was numerically higher in subgroups of patients with higher tumor PD-L1 expression and in the subgroup of patients with ER positivity <10% (who have previously been reported to have biological characteristics and clinical outcomes similar to those of patients with TNBC^11–14^). However, these results should be interpreted with caution as these subgroups are underpowered, and the only objective of subgroup analyses is to explore convergent validity. Post hoc exploratory subgroup analyses of pCR based on PD-L1 CPS of ≥20 and based on both PD-L1 CPSs and ER positivity were reported. It is important to exercise caution when interpreting these observations, especially with very small sample sizes. To establish confidence, replication in other trials is absolutely essential. The efficacy associated with neoadjuvant pembrolizumab‒chemotherapy was supported by the similar results observed when defined per secondary endpoints (that is, ypT0 ypN0 and ypT0/Tis). The addition of pembrolizumab to neoadjuvant chemotherapy also shifted more patients to lower RCB categories (RCB-0 and RCB-1), demonstrating the ability of the combination to reduce tumor tissue remaining after surgery among those without pCR. Reduced RCB has been associated with improved EFS^15^.

Results from our study are consistent with those from the phase 2 I-SPY2 study in which the addition of pembrolizumab to chemotherapy improved pCR in patients with previously untreated, high-risk, early-stage, HER2^−^ breast cancer^8^. In the phase 3 CheckMate 7FL study, a similar improvement in pCR outcomes was reported among patients with high-risk, early-stage, grade 2/grade 3 ER^+^/HER2^−^ breast cancer who received nivolumab plus neoadjuvant chemotherapy compared to those who received placebo plus neoadjuvant chemotherapy (24.5% versus 13.8%; odds ratio = 2.05 (95% CI, 1.29‒3.27); *P* = 0.0021)^16^.

In the adjuvant phase of our study, patients received pembrolizumab or placebo plus standard endocrine therapy. The monarchE study demonstrated that the addition of the cyclin-dependent kinase 4/cyclin-dependent kinase 6 inhibitor abemaciclib to adjuvant endocrine therapy improved relapse-free survival in patients with hormone receptor-positive, HER2^−^, node-positive, high-risk, early-stage breast cancer^17^. However, the present study was designed before these results were reported, and the use of adjuvant abemaciclib was not permitted per protocol. Given that pCR was assessed before the adjuvant phase, the use of abemaciclib could not have influenced this outcome. Potential safety risks have been reported in studies investigating cyclin-dependent kinase 4/6 inhibitor plus immune checkpoint inhibitor combination therapy in patients with hormone receptor-positive, HER2^−^ metastatic breast cancer^18–20^. Current guidelines also do not recommend such combination therapy^21^.

Data for the study’s other primary endpoint of EFS are not mature and continue to be evaluated. The EFS outcome will reflect the overall treatment regimen effect, including both the neoadjuvant and adjuvant treatment phases. This study was not designed to discriminate the relative contribution of each phase; a prospective trial would be needed to address this question.

AEs in the pembrolizumab‒chemotherapy arm were consistent with the known safety profiles of the individual agents. Similar to results from the KEYNOTE-522 study (which evaluated pembrolizumab in combination with neoadjuvant chemotherapy among patients with TNBC)^9,10^, there was no evidence that the addition of pembrolizumab to standard neoadjuvant chemotherapy exacerbated chemotherapy-associated toxicity, and the most frequently occurring events in both arms were those typically associated with cytotoxic chemotherapy, such as alopecia, nausea and anemia. Incidences of treatment-related grade ≥3 AEs and treatment-related AEs leading to treatment discontinuation were moderately higher with pembrolizumab‒chemotherapy than with placebo‒chemotherapy. As anticipated based on prior studies evaluating anti-PD-1 and anti-PD-L1 monoclonal antibodies^22^, AEs with a potentially immune-mediated mechanism occurred more frequently among patients in the pembrolizumab‒chemotherapy arm than in the placebo‒chemotherapy arm. Broadly, the safety profile in this study was consistent with that in the KEYNOTE-522 study^9,10^.

In conclusion, neoadjuvant pembrolizumab‒chemotherapy resulted in an improved pCR rate compared with chemotherapy alone in patients with high-risk, early-stage, ER^+^/HER2^−^ breast cancer. Evaluation of EFS is ongoing.

## Methods

### Patients

Eligible patients were ≥18 years of age with centrally confirmed ER^+^/HER2^−^ grade 3 invasive ductal breast carcinoma (evaluated according to the most recent guidelines of the American Society of Clinical Oncology–College of American Pathologists^23,24^); newly diagnosed, previously untreated, nonmetastatic disease (T1c–2 (≥2 cm), cN1–2 or T3–4, cN0–2, per the most current staging criteria of the American Joint Committee on Cancer (AJCC)), as determined by the investigator in radiologic assessment, clinical assessment or both; an Eastern Cooperative Oncology Group (ECOG) performance-status score ≤1; and adequate organ function. Patients with multifocal primary tumors, inflammatory breast cancer and those with low ER positivity were eligible.

Exclusion criteria included active bilateral, multicentric, lobular or metastatic breast cancer; autoimmune disease for which the patient was treated with systemic therapy within 2 years; diagnosis of immunodeficiency or use of immunosuppressive therapy within 1 week; history of noninfectious pneumonitis treated with steroids; pneumonitis; active tuberculosis; active infection for which the patient was receiving systemic therapy; or clinically significant cardiovascular disease. Full eligibility criteria are listed in the Supplementary Information.

### Trial design and treatment

In this randomized, double-blind trial (ClinicalTrials.gov identifier, NCT03725059), patients received treatment in neoadjuvant and adjuvant phases. No crossover between treatment arms was permitted between the phases. Based on emerging clinical data in patients with early-stage breast cancer demonstrating heterogeneous long-term clinical outcomes across countries/regions despite similar standard of care treatments, patients were stratified at randomization by region (Eastern Europe versus China versus all other countries) to ensure balance across treatment arms. Patients from Eastern Europe were further stratified by tumor PD-L1 status (CPS ≥ 1 versus < 1). Patients from China were not further stratified. Patients from all other countries (that is, excluding Eastern Europe and China) were further stratified by tumor PD-L1 status (CPS ≥ 1 versus < 1), lymph node involvement (positive versus negative), anthracycline dosing schedule (Q2W versus Q3W) and ER positivity (≥10% versus <10%). Patients were randomly assigned (in a 1:1 ratio) to the pembrolizumab–chemotherapy arm or the placebo–chemotherapy arm using a central interactive voice-response system with an integrated web-response system. In the neoadjuvant phase, patients received four cycles of intravenous pembrolizumab (200 mg) or placebo once Q3W plus paclitaxel QW (80 mg m^−^^2^; first neoadjuvant treatment), followed by four cycles of pembrolizumab or placebo in combination with either doxorubicin (60 mg m^−^^2^) or epirubicin (100 mg m^−2^) plus cyclophosphamide (600 mg m^−^^2^) administered either Q2W or Q3W (second neoadjuvant treatment). Patients who either completed or discontinued the first neoadjuvant treatment could start the second neoadjuvant treatment or undergo surgery, and those who completed or discontinued the second neoadjuvant treatment could undergo surgery. Patients underwent surgery (breast conservation or mastectomy with/without sentinel lymph node biopsy or axillary dissection) no later than 6 weeks after the last dose of the neoadjuvant treatment. In the adjuvant phase, patients received (beginning within 60 days after surgery) pembrolizumab or placebo Q3W for up to nine cycles (up to 6 months), plus the investigator’s choice of endocrine therapy (per institutional guidelines) for up to 10 years. Adjuvant therapy with abemaciclib was not permitted. Adjuvant radiation therapy (per institutional guidelines) was permitted, as indicated, either before initiation of adjuvant therapy or concurrently. Trial treatment was discontinued in patients with disease progression or recurrence or unacceptable toxic effects.

The study was developed by a scientific advisory committee and employees of the sponsor (Merck Sharp & Dohme LLC, a subsidiary of Merck & Co., Inc., Rahway, NJ, USA). An external independent data monitoring committee oversaw the study, periodically assessed safety, and assessed efficacy at prespecified interim analyses. The protocol was approved by an ethics body at each study site (see Supplementary Information for a list of study sites). Patients provided written informed consent

### Assessments

Upon completion of neoadjuvant therapy, pCR was assessed according to the AJCC staging criteria (ypT0/Tis ypN0, ypT0 ypN0 and ypT0/Tis) by a local pathologist blinded to treatment assignment. EFS (defined as the time from randomization to disease progression that precluded surgery, local or distant recurrence, second primary cancer or death due to any cause, whichever occurred first) was evaluated in a blinded fashion by the investigator. PD-L1 expression in new or recent core needle biopsy samples was assessed at a central laboratory using PD-L1 IHC 22C3 pharmDx (Agilent Technologies). AEs were assessed throughout the trial and for 30 days after discontinuation of treatment (90 days for serious AEs) and graded according to Common Terminology Criteria for AEs (v4.0)^25^. Immune-mediated AEs were based on a list of preferred terms intended to capture known risks of pembrolizumab and were considered regardless of attribution to study treatment by the investigator.

### Endpoints

The study’s primary endpoints were pCR, defined as ypT0/Tis ypN0 at the time of surgery, and EFS in the intention-to-treat population. Secondary endpoints included pCR according to the definitions of ypT0 ypN0 and ypT0/Tis in all patients, pCR according to all definitions in patients with PD-L1 CPS ≥ 1 tumor, EFS among patients with PD-L1 CPS ≥ 1 tumor and OS among all patients and patients with PD-L1 CPS ≥ 1 tumor. Safety during the neoadjuvant and adjuvant phases was evaluated in all patients who received ≥1 trial drug, underwent surgery or both. Evaluation of RCB (residual disease in either the breast or lymph node at the time of surgery) was an exploratory endpoint.

### Statistical analysis

Efficacy was evaluated in the intention-to-treat population, which included all patients who had undergone randomization. Safety was evaluated in the as-treated population, which included all patients who had undergone randomization and received ≥1 trial drug, underwent surgery or both. We used the stratified Miettinen–Nurminen method^26^, with weights proportional to the stratum sample size, to compare between-arm differences in percentages of patients with a pCR. Patients who did not have pCR results for any reason or who received neoadjuvant treatment not specified in the protocol were considered as not having a response.

The 95% CIs associated with the between-arm differences in the percentages of patients with a pCR were not adjusted for multiple comparisons and therefore cannot be used to infer effects. The stratification factors used at randomization with the prespecified pooling strategy were used in all stratified analyses.

**Extended Data Fig. 2:**
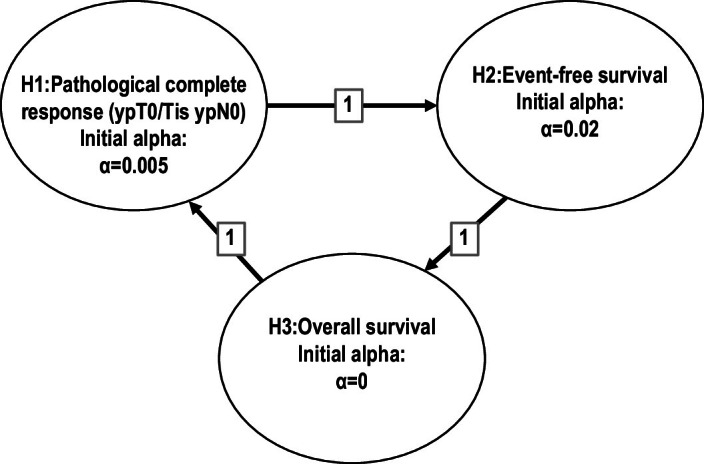
Multiplicity graph for type I error control of study hypotheses. The prespecified analysis plan allows alpha passing from successful endpoint(s) to other(s). H, hypothesis.

The graphical method discussed in ref. ^27^ was applied to control the type I error rate at a one-sided *α* level of 0.025 across both primary endpoints and all interim and final analyses (Extended Data Fig. 2). The Lan–DeMets O’Brien–Fleming spending function was used to control the type I error in the interim and final analyses. The primary purpose of the first interim analysis was to evaluate the superiority of pembrolizumab–chemotherapy over placebo–chemotherapy with respect to the percentage of patients with a pCR (stage ypT0/Tis, ypN0); this analysis was to occur after enrollment was completed and all randomized patients would have had surgery after approximately 6 months of neoadjuvant treatment.

With an enrollment of approximately 1,240 patients, the trial had >99% power to detect a true difference of 15 percentage points for the comparison of the rate of pCR (stage ypT0/Tis ypN0) between the treatment arms at a one-sided *α* level of 0.005. It would have a power of 84% to detect a hazard ratio for EFS of 0.73 at a one-sided *α* level of 0.02 at the final analysis.

Statistical analyses were conducted using SAS v9.4 (SAS Institute).

### Reporting summary

Further information on research design is available in the Nature Portfolio Reporting Summary linked to this article.

## Online content

Any methods, additional references, Nature Portfolio reporting summaries, source data, extended data, Supplementary Information, acknowledgements, peer review information; details of author contributions and competing interests; and statements of data and code availability are available at 10.1038/s41591-024-03415-7.

## Supplementary information

Supplementary Information List of investigators and study sites, steering committee, data monitoring committee and eligibility criteria.

Reporting Summary

## Figures and Tables

**Extended Data Table 1:**
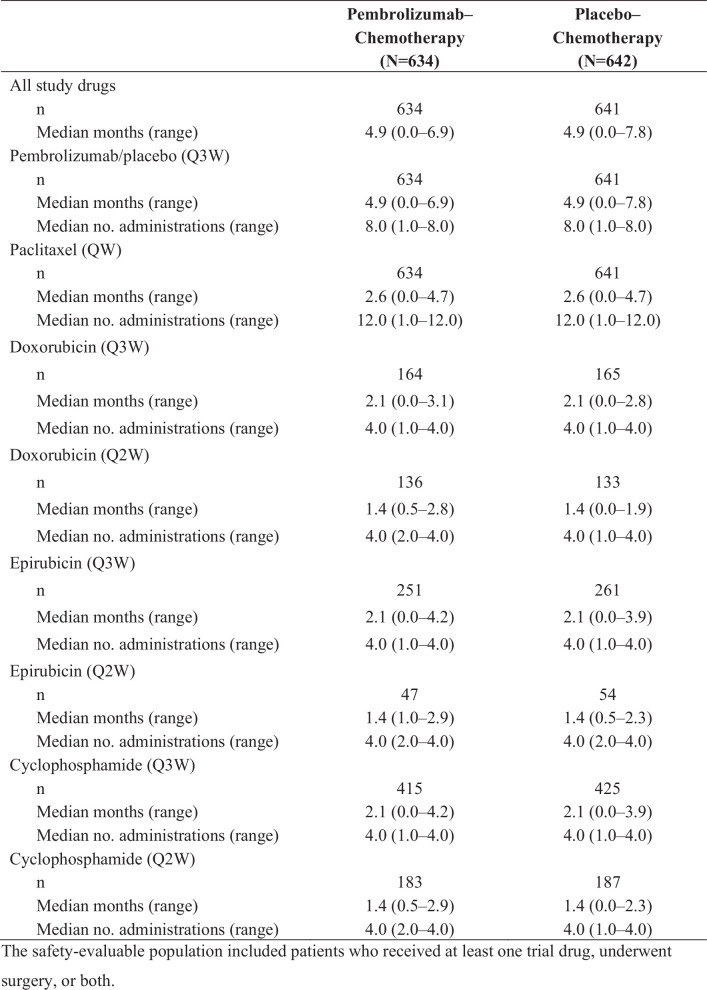
Summary of drug exposure during the neoadjuvant phase

## Data Availability

Merck Sharp & Dohme LLC, a subsidiary of Merck & Co., Inc., Rahway, NJ, USA (MSD), is committed to providing qualified scientific researchers access to anonymized data and clinical study reports from the company’s clinical trials for the purpose of conducting legitimate scientific research. MSD is also obligated to protect the rights and privacy of trial participants and, as such, has a procedure in place for evaluating and fulfilling requests for sharing company clinical trial data with qualified external scientific researchers. The MSD data-sharing website (available at engagezone.msd.com/ds_documentation.php) outlines the process and requirements for submitting a data request. Applications will be promptly assessed for completeness and policy compliance. Feasible requests will be reviewed by a committee of MSD subject matter experts to assess the scientific validity of the request and the qualifications of the requestors. In line with data privacy legislation, submitters of approved requests must enter into a standard data-sharing agreement with MSD before data access is granted. Data will be made available for request after product approval in the United States and European Union or after product development is discontinued. There are circumstances that may prevent MSD from sharing requested data, including country- or region-specific regulations. If the request is declined, it will be communicated to the investigator. Access to genetic or exploratory biomarker data requires a detailed, hypothesis-driven statistical analysis plan that is collaboratively developed by the requestor and MSD subject matter experts; after approval of the statistical analysis plan and execution of a data-sharing agreement, MSD will either perform the proposed analyses and share the results with the requestor or will construct biomarker covariates and add them to a file with clinical data that is uploaded to an analysis portal so that the requestor can perform the proposed analyses.
