# Supplementary material for: Pembrolizumab and chemotherapy in high-risk, early-stage, ER+/HER2− breast cancer: a randomized phase 3 trial
Source: Nat Med. 2025 Jan 21;31(2):442–8. doi: 10.1038/s41591-024-03415-7 (PMC11835712; doi:10.1038/s41591-024-03415-7)
Supplement: Supplementary file 1 — List of investigators and study sites, steering committee, data monitoring committee and eligibility criteria. [file 41591_2024_3415_MOESM1_ESM.pdf]

nature medicine

Supplementary information https://doi.org/10.1038/s41591-024-03415-7

Pembrolizumab and chemotherapy in high-risk, early-stage, $ER^+/HER2^-$ breast cancer: a randomized phase 3 trial

In the format provided by the authors and unedited

SUPPLEMENTARY INFORMATION

List of Investigators and Study Sites

Country	Site Name	Principal Investigator
Australia	Royal North Shore Hospital	Menzies, Alexander
	Westmead Hospital	Dinh, Phuong
	Peter MacCallum Cancer Centre	Loi, Sherene
	Frankston Hospital	Thomson, Jacqui
	St. John of God Subiaco Hospital	Tsoi, Daphne
	Mater Misericordiae Ltd	Middleton, Kathryn
	Chris O'Brien Lifehouse	Beith, Jane
Belgium	AZ Maria Middelares Gent	Vulsteke, Christof
	Cliniques Universitaires Saint-Luc	Duhoux, Francois
	UZ Leuven	Schöffski, Patrick
	Imelda Ziekenhuis Bonheiden	Van den Bulck, Heidi
	Jessa Ziekenhuis Campus Virga Jesse	Mebis, Jeroen
	AZ Groeninge Hospital	Borms, Marleen
	CHU UCL Namur Site de Godinne	D Hondt, Lionel
	CHC MontLégia	Graas, Marie-Pascale
	AZ Sint-Jan Brugge	Claes, Nele
	UZ Antwerpen - Medical Oncology	Altintas, Sevilay
	Institut Jules Bordet	Gombos, Andrea
Brazil	INCA HC III - Instituto Nacional de Câncer	Bines, Jose
	Associacao Hospitalar Moinhos de Vento	Morelle, Alessandra
	União Brasileira de Educação e Assistência Hospital São Lucas da PUCRS	Werutsky, Gustavo
	Ensino e Terapia de Inovacao Clinica AMO	Dybal, Vanessa
	Clinica de Pesquisas e Centro de Estudos em Oncologia Ginecologica e Mamaria Ltda	Hegg, Roberto
	Hospital Araujo Jorge Associacao de Combate ao Cancer de Goias	Freitas Junior, Ruffo de
	ONCOSITE - Centro de Pesquisa Clinica em Oncologia	Franke, Fabio
	Centro de Novos Tratamentos Itajai - Clinica de Neoplasias Litoral	Borges, Giuliano
	CEPON - Centro de Pesquisas Oncológicas	Matsuda, Antonio Jose Carvalho
	ICESP – Instituto do Câncer do Estado de São Paulo	Linck, Rudinei Diogo Marques
	Núcleo de Pesquisa Clínica da Rede São Camilo	Cruz, Felipe José
Canada	North York General Hospital	Yu, Joanne
	CHU de Quebec Universite Laval - Hopital du Saint-Sacrement	Poirier, Brigitte
	Moncton Hospital - Horizon Health Network	Harb, Mohammed

	Jewish General Hospital	Boileau, Jean-Francois
	CIUSSS de l'Estrie-CHUS	Pavic, Michel
	Saskatoon Cancer Centre	Haider, Kamal-Uddin/Iqbal, Nayyer
	Centre Hospitalier Régional de Trois-Rivières	Aucoin, Jean Sebastien
	Cancer Centre of Southeastern Ontario, Kingston General Hospital	Robinson, Andrew
	CISSS de la Montérégie-Centre	Prady, Catherine
	CancerCare Manitoba	Kim, Christina
	Nova Scotia Health Authority	Drucker, Arik
	Maisonneuve-Rosemont Hospital	Sideris, Lucas
	Princess Margaret Cancer Centre	Cescon, David
	Ottawa Hospital Research Institute	Song, Xinni
	CHUM - Centre Hospitalier de l'Université de Montréal	Younan, Rami
	Cross Cancer Institute	Basi, Sanraj
	BC Cancer - Vancouver Center	Chia, Stephen
	Sunnybrook Research Institute	Pezo, Rossanna
China	Harbin Medical University Cancer Hospital	Pang, Da
	The First Hospital of Jilin University	Fan, Zhimin
	West China Hospital of Sichuan University	Jing, Jing
	The First Affiliated Hospital of Zhejiang University	Fu, Peifen
	Guangdong General Hospital	Liao, Ning
	Fudan University Shanghai Cancer Center	Shao, Zhimin
	Renji Hospital Shanghai Jiaotong University School of Medicine	Xu, Shuguang
	Fujian Medical University Union Hospital	Zhang, Jie
	Cancer Hospital Chinese Academy of Medical Sciences	Wang, Jing
	Tianjin Medical University Cancer Institute & Hospital	Zhang, Jin
	Zhejiang Cancer Hospital	Yang, Hongjian
	Hubei Cancer Hospital	Wu, Xinhong
	Henan Cancer Hospital	Liu, Zhenzhen
	Sun Yat-Sen Memorial Hospital of Sun Yat-Sen University	Liu, Qiang
	Hunan Cancer Hospital	Ouyang, Quchang
	Ruijin Hosp, Shanghai Jiao Tong University School of Medicine	Shen, Kunwei
	Fourth Hospital of Hebei Medical University	Geng, Cuizhi
	Changhai Hospital of Shanghai	Sheng, Yuan
	Cancer Hospital Affiliated to Xinjiang Medical University	Ou, Jianghua
	The First Affiliated Hospital of Xi an Jiaotong University	Ren, Yu
	Shenzhen Second People's hospital	Wang, Xianming

	Anhui Provincial Hospital	Ma, Xiaopeng
	Zhejiang Provincial People's Hospital	Meng, Xuli
Colombia	Clinica de la Costa Ltda	Alcala Castro, Carmen
	Oncomedica S.A.	Gonzalez Fernandez, Manuel
	Centro de Investigacion Clínica del Country	Niño Gomez, Oscar
	Fundacion Universitaria Sanitas	Garcia, Diego
	Fundación Valle del Lili	Restrepo, Juan
	Clínica Vida Fundación - Sede Poblado	Llinas Quintero, Nestor
	Centro Medico Imbanaco de Cali S.A	Urrego Melendez, Olga
	Rodrigo Botero SAS	Salinas, Jorge
	Fundación Cardiovascular de Colombia	Forero Moreno, Elkin
Costa Rica	CIMCA Centro de Investigación y Manejo del Cáncer	Corrales, Luis
	Onco Tech SA	Gonzalez Herrera, Ileana
	ICIMED - Instituto de Investigacion en Ciencias Medicas	Guzman Ramirez, Adrian
	Hospital Metropolitano - Sede Lindora	Wiernik Rodriguez, Andres
France	Institut Curie; Institut Curie Paris and Saint Cloud	Loirat, Delphine
	Institut Claudius Regaud IUCT Oncopole	Dalenc, Florence
	Clinique Victor Hugo	BOURGEOIS, Hugues
	Hopital prive du Confluent	Chocteau-bouju, Dorothee
	Hôpital Saint-Louis	Giacchetti, Sylvie
	Centre Jean Perrin	Mouret-Reynier, Marie-Ange
	Centre Eugène Marquis	Le Du, Fanny
	Centre Oscar Lambret	Hajjaji, Nawale
	Centre Hospitalier Annecy Genevois	Stefani, Laetitia
	Hôpital Tenon	Gligorov, Joseph
	CHU AMIENS Sude-Salouel	Bihan, Celine
	Institut Sainte Catherine	Billemont, Bertrand
	Centre Paul Strauss	Petit, Thierry
	CHR Metz-Thionville - Hôpital de Mercy	Luporsi, Elisabeth
	Centre Georges Francois Leclerc	Desmoulins, Isabelle
	Hopital Europeen Marseille	Dalivoust, Philippe
	Institut de Cancerologie du Gard - CHU Caremeau	FITENI, Frédéric
	Centre D Oncologie de Gentilly	Spaeth, Dominique
	Centre de Cancérologie du Grand Montpellier	Villanueva, Cristian
	Institut Gustave Roussy	Andre, Fabrice
	Centre François Baclesse	Levy, Christelle
Germany	Klinikum der Universitaet Muenchen - Grosshadern	Wuerstlein, Rachel/Koenig, Alexander
	Universitätsklinikum Erlangen	Fasching, Peter
	Sana Klinikum Offenbach GmbH	Khodaverdi, Silvia/Jackisch, Christian
	Gynäkologisches Zentrum	Kurbacher, Christian

	HELIOS Dr. Horst Schmidt Kliniken Wiesbaden	Eichbaum, Michael
	MVZ Nordhausen gGmbH - Praxis Dr. Grafe	Grafe, Andrea
	Kliniken Essen-Mitte GmbH Evang. Huyssens Stiftung	Kümmel, Sherko
	Universitaetsklinikum Ulm	Huober, Jens
	Universitätsklinikum Carl Gustav Carus	Wimberger, Pauline
	Caritas Klinikum Saarbrücken St. Theresia	Deryal, Mustafa
	Private Clinic - Dr. Gerhard Klausmann	Klausmann, Martine
	VKK Studien GbR	Rieger, Lorenz
	Medizinische Management GmbH	Mayer, Frank
	Gynäkologisch-onkologische Praxis Hannover	Lueck, Hans Joachim
	Frauenklinik St. Louise	Wuellner, Michaela/Lux, Michael
Hungary	Orszagos Onkologiai Intezet	Bittner, Nóra/Rubovszky, Gabor
	Szent Margit Korhaz	Boer, Katalin
	Uzsoki Utcai Korhaz	Meszaros, Edina
	Somogy Megyei Kaposi Mor Oktato Korhaz	Ruzsa, Agnes/Balint, Andras
	Borsod-Abaúj-Zemplén Megyei Kórház és Egyetemi Oktatókórház	Solymosi, Tibor
	Pecsi Tudomanyegyetem Klinikai Kozpont	Mangel, László Csaba
	Szegedi Tudomanyegyetem - Szent Györgyi Albert Klinikai Központ	Kahan, Zsuzsanna
	Debreceni Egyetem Klinikai Kozpont	Arkosy, Peter
	Semmelweis Egyetem	Dank, Magdolna
	Pest Megyei Flor Ferenc Korhaz	Kammerer, Kinga
	Dr. Bugyi István Kórház	Kispal, Mihaly
	Bacs-Kiskun Megyei Korhaz	Horvath, Zsolt
	Zala Megyei Szent Rafael Korhaz	Mahr, Karoly
Ireland	St Vincent's Hospital	Gullo, Giuseppe
	Mater Misericordiae University Hospital	Higgins, Michaela
	Saint James's Hospital	O Hanlon Brown, Ciara
	Bon Secours Hospital	Murphy, Conleth
Israel	Hadassah Ein Karem - Sharett Institute of Oncology	Peretz-Yablonski, Tamar
	Soroka Medical Center	Tokar, Margarita
	Rabin Medical Center	Stemmer, Salomon
	Kaplan Medical Center	Ben Baruch, Noa
	Assuta Ashdod Public	Ryvo, Larisa
	Rambam Health Care Campus - Oncology Division	Shai, Ayelet
	Holy Family Hospital	Mhameed, Kamel
	Sourasky Medical Center	Sonnenblick, Amir
	Chaim Sheba Medical Center	Nili-Gal-Yam, Einav
	Shaare Zedek Medical Center	Gabizon, Alberto
	Assuta Medical Center	Jiveliouk, Irina

	Meir Medical Center	Kuchuk, Iryna
	HaEmek Medical Center	Bar-Sela, Gil
Japan	Hyogo College of Medicine Hospital	Miyoshi, Yasuo
	Aichi Cancer Center Hospital	Iwata, Hiroji
	Kumamoto University Hospital	Yamamoto, Yutaka
	Hiroshima City Hiroshima Citizens Hospital	Itoh, Mitsuya
	The Cancer Institute Hospital of JFCR	Takano, Toshimi
	Chiba Cancer Center	Yamamoto, Naohito
	Saitama Medical University International Medical Center	Osaki, Akihiko
	National Hospital Organization Hokkaido Cancer Center	Maeda, Hideki
	Toranomon Hospital	Tanabe, Yuko
	Social medical corporation Hakuaikai Sagara Hospital	Sagara, Yasuaki
	Fukushima Medical University Hospital	Saji, Shigehira
	Shizuoka Cancer Center Hospital and Research Institute	Tadokoro, Yukiko
	Saitama Cancer Center	Inoue, Kenichi
	National Cancer Center Hospital East	Mukohara, Toru
	National Hospital Organization - Osaka National Hospital - Institute For Clinical Research	Yasojima, Hiroyuki
	Showa University Hospital	Hayashi, Naoki
	Kitasato University Hospital	Sangai, Takafumi
New Zealand	Capital & Coast District Health Board - Wellington Hospital	Barton, Sarah
	Tauranga Hospital	Jones, Joanna
	Canterbury Regional Cancer & Blood Services	McLachlan, Jennifer
Poland	Centrum Onkologii im. Prof. Franciszka Lukaszczyka	Zurawski, Bogdan
	Narodowy Instytut Onkologii - Oddzial w Gliwicach	Tarnawski, Rafal
	Wojewódzki Szpital Zespolony im. L. Rydygiera w Toruniu	Sawrycki, Piotr
	Mazowiecki Szpital Onkologiczny	Bauer-Kosinska, Barbara
	Szpital Wojewódzki Koszalinie im. Mikolaja Kopernika	Kwiatkowski, Mariusz
	Uniwersyteckie Centrum Kliniczne Slaskiego Uniwersytetu Medycznego	Szablowska-Siwik, Sylwia
	Dolnoslaskie Centrum Onkologii.	Lacko, Aleksandra/Soter, Katarzyna
	Wojewodzki Szpital Specjalistyczny nr 4 w Bytomiu	Nowakowska-Zajdel, Ewa
	Beskidzkie Centrum Onkologii im. Jana Pawla II	Kowalski, Marcin
	Osrodek Badan Klinicznych Gyncentrum	Kalmuk, Andrzej

	Narodowy Instytut Onkologii im. Marii Sklodowskiej-Curie	Grela-Wojewoda, Aleksandra
	Mazowiecki Szpital Specjalistyczny im. dr Józefa Psarskiego	Domurad, Agnieszka
	Samodzielny Publiczny Szpital Kliniczny Nr 1 w Lublinie	Polkowski, Wojciech
	Wojewodzkie Centrum Onkologii Copernicus	Wojcik-Tomaszewska, Joanna
	Szpitale Pomorskie Sp. z o.o.	Danielewicz, Iwona
	Bialostockie Centrum Onkologii	Wojtukiewicz, Marek
	Instytut Centrum Zdrowia Matki Polki	Kalinka, Ewa
	Narodowy Instytut Onkologii im. Marii Skłodowskiej-Curie - Panstwowy Instytut Badawczy w Warszawie	Nowecki, Zbigniew
Portugal	Champalimaud Clinical Center/Champalimaud Foundation	Cardoso, Fatima
	CHLN Hospital Santa Maria	Ribeiro, Leonor
	Inst. Portugues de Oncologia de Porto Francisco Gentil EPE	Ferreira, Marta
	Hospital Geral de Santo Antonio	Simoes, Joana
Puerto Rico	UPR Comprehensive Cancer Center	Mora, Edna
	Centro De Cancer De La Mujer	Di Marco, Anna
Russia	Scientific Research Oncology Institute n.a. N.N.Petrov	Krivorotko, Petr
	Clinical Research Center of specialized types medical care - Oncology	Moiseyenko, Vladimir
	Arkhangelsk Clinical Oncological Dispensary	Chapko, Yana
	Republican Clinical Oncology Dispensary of Tatarstan MoH	Mukhametgaleeva, Farida
	Altay Regional Oncology Dispensary	Lazarev, Sergey
	Tomsk Scientific Research Institute of Oncology	Usynin, Evgeniy
	Novosibirsk Regional Clinical Oncology Dispensary	Kozlov, Vadim
	Central Clinical Hospital with outpatient Clinic	Nosov, Dmitry
	N.N. Blokhin NMRCO	Frolova, Mona
	Republican Clinical Oncology Dispensary of Republic of Bashkortostan	Lipatov, Oleg
	Ryazan Regional Clinical Oncology Dispensary	Shomova, Marina
	Leningrad Regional Oncology Center	Vats, Anna
	Medical Rehabilitation Center	Belonogov, Aleksandr
	Railway Hospital of OJSC	Vasiliev, Aleksandr
South Korea	Seoul National University Hospital	Im, Seock-Ah
	Severance Hospital Yonsei University Health System	Sohn, Joo Hyuk
	Asan Medical Center	Kim, Sung-Bae
	Samsung Medical Center	Park, Yeon Hee
	National Cancer Center	Lee, Keun Seok

Spain	Hospital Ruber Internacional	Gión Cortes, Maria
	Hospital Universitario Ramon y Cajal	Holgado Martin, Esther
	Hospital Quiron de Madrid	Gonzalez Cortijo, Lucia
	Instituto Oncologico Baselga.Hospital Quiron	Soberino Garcia, Jesus
	Hospital Clinic I Provincial de Barcelona	Pascual Martinez, Tomas
	Hospital Clinico San Carlos	Garcia Saenz, Jose
	Hospital Clínico Universitario de Valencia	Bermejo de las Heras, Begona
	Hospital Universitario 12 de Octubre	Ciruelos Gil, Eva
	Hospital Vall D Hebron	Zamora Adelantado, Esther
	Hospital Teresa Herrera - Chuac	Antolin Novoa, Silvia
	Hospital Clinico Universitario de Salamanca	Rodriguez Sanchez, Cesar
	Hospital Universitario Virgen del Rocio	Salvador Bofill, Javier
	Hospital Virgen de las Nieves	Gonzalez Flores, Encarnacion
	Hospital Universitario Insular de Gran Canaria	Vicente Rubio, Elena
	Instituto Catalan de Oncologia ICO - Hospital Duran i Reynals	Gil Gil, Miguel
	Complejo Hospitalario de Jaen	Sanchez Rovira, Pedro
	Hospital Universitario La Fe	Santaballa Bertran, Ana
	Hospital General Universitario Gregorio Maranon	Martin Jimenez, Miguel
	Hospital Clinico San Cecilio	Blancas, Isabel
	Hospital General Arnau de Vilanova de Valencia	Llombart Cussac, Antonio
Taiwan	National Cheng Kung University Hospital	Lee, Kuo-Ting
	China Medical University Hospital	Wang, Hwei-Chung
	Linkou Chang Gung Memorial Hospital	Chen, Shin-Cheh
	Koo Foundation Sun Yat-Sen Cancer Center	Chung, Chi-Feng
	National Taiwan University Hospital	Huang, Chiun-Sheng
United Kingdom	Barts Health NHS Trust	Schmid, Peter
	Guy's Hospital	Mansi, Janine
	Royal Cornwall Hospital	Wheatley, Duncan
	University Hospitals Bristol NHS Foundation Trust	Braybrooke, Jeremy
	Nottingham University Hospitals NHS Trust	Chan, Steve
	St. Georges University Hospital NHS Foundation Trust	Kyle, Fiona
	Birmingham & Solihull Heartlands Hospital NHS	Tsalic, Medy
	The Clatterbridge Cancer Centre NHS Foundation Trust	Palmieri, Carlo
	Colchester General Hospital	Mukesh, MB
United States	Cancer Treatment Centers of America at Western Regional Medical Center	Lynch, Cynthia
	University of Arizona Cancer Center	Gordon, Michael
	University of Arizona	Chalasani, Pavani

	El Camino Hospital Cancer Center	Li, Jiali
	Pacific Cancer Care	Stampleman, Laura
	Providence Health/John Wayne Cancer Institute	ODay, Steven
	University of California - San Diego	Helsten, Teresa
	University of Colorado Cancer Center	Diamond, Jennifer
	Yale University School of Medicine	Pusztai, Lajos
	Florida Hospital Cancer Institute	Alemany, Carlos
	Memorial Cancer Institute	Perez, Alejandra
	University of Miami Sylvester CC	Valdes, Frances
	H. Lee Moffitt Cancer Center and Research Institute	Han, Hyo
	Baptist MD Anderson Cancer Center	Zuberi, Lara
	Holy Cross Hospital-Fort Lauderdale	Segota, Zdenka
	MercyOne Waterloo Cancer Center	Masri, Mohammed
	NorthShore University Health System	Saha, Poornima
	Robert H Lurie CCC, Feinburg School of Medicine	Cristofanilli, Massimo
	Northwestern University Feinberg School of Medicine	Gradishar, William
	Orchard Healthcare Research Inc.	Oliff, Ira
	Goshen Center for Center Care	Kio, Ebenezer
	James Graham Brown Cancer Center	Riley, Elizabeth
	Mary Bird Perkins Cancer Center at St. Tammany Parish Hospital	Spell, Derrick
	Massachusetts General Hospital	Bardia, Aditya
	Univ. of Massachusetts Memorial Medical Center-University Campus	Toke, Madhavi
	Johns Hopkins University	Wolff, Antonio
	Barbara Ann Karmanos Cancer Institute	Assad, Hadeel
	Henry Ford Health System	Ali, Haythem
	Mayo Clinic and Medical School (Rochester)	Liu, Minetta
	Washington University School of Medicine	Hernandez Aya, Leonel
	Saint Luke's Health System	Pluard, Timothy
	Glacier View Research Institute	Hattersley Anderes, Elise
	St. Vincent Frontier Cancer Center	Cobb, Patrick
	Wake Forest School of Medicine	Thomas, Alexandra
	Duke University Medical Center	Marcom, Kelly
	UNC Hospitals	Carey, Lisa
	Sanford Roger Maris Cancer Center	Panwalkar, Amit
	Southeast Nebraska Cancer Center	Berg, Alan
	Oncology Hematology West, PC dba Nebraska Cancer Specialists	Block, Margaret
	University of Nebraska Medical Center	Krishnamurthy, Jairam
	Holy Name Medical Center	Rivera, Yadyra
	University of New Mexico	Brown-Glaberman, Ursa
	Weill Cornell Medical College	Andreopoulou, Eleni
	Hematology-Oncology Associates of Rockland	Rybalova, Irina

	Prohealthcare Associates, LLP	Citron, Marc
	Cleveland Clinic	Montero, Albert
	Hematology & Oncology Associates, Inc.	Haut, Mitchell
	Tri-County Hematology & Oncology Associates, Inc.	Rafique, Noman
	Ohio State University Arthur G James Cancer Hospital & Richard J Solove Research Institute	Wesolowski, Robert
	Providence Portland Medical Center	Page, David
	OHSU Knight Cancer Institute	Hobbs, Evthokia
	Geisinger Medical Center	Ramdin, Nadia
	Medical University of South Carolina	Brescia, Frank
	Avera Cancer Institute	Krie, Amy
	Sanford Cancer Center Oncology Clinic	Sanford, Amy
	West Clinic Comprehensive Breast Center	Vidal, Gregory
	Vanderbilt University	Abramson, Vandana
	University of Texas Southwestern Medical Center at Dallas	Haley, Barbara
	Houston Methodist Hospital System	Chang, Jenny
	Baylor College of Medicine	Rimawi, Mothaffar
	Huntsman Cancer Institute	Cohen, Adam
	Intermountain Medical Center	Van Meter, Margaret
	University of Virginia	Dillon, Patrick
	Bon Secours St. Francis Medical Center Oncology Research	Irvin, William
	University of Vermont	Dittus, Kim
	Medical Oncology Associates	Chaudhry, Arvind
	Cancer Care Northwest	Martincic, Danko
	Northwest Medical Specialties, PLLC	Senecal, Francis
	Seattle Cancer Care Alliance/University of Washington Medical Center	Specht, Jennifer
	Kadlec Clinic Hematology and Oncology	Zhuo, Ying
	Rutgers Cancer Institute of New Jersey	Toppmeyer, Deborah
	Stanford Cancer Center	Telli, Melinda
	UC Davis Comprehensive Cancer Center	Chew, Helen
	CTCA Southwestern	Karippot, Asha
	Southeastern Regional Medical Center, Inc.	Hansra, Damien
	Cancer Treatment Centers of America-Eastern Regional Medical Center	Aithal, Sramila
	Midwestern Regional Medical Center, Inc.	Ahn, Eugene
	Fox Chase Cancer Center	Jain, Angela
	Cedars Sinai Medical Center Samuel Oschin Comp. Cancer Institute	McArthur, Heather
	The University of Chicago Medical Center	Nanda, Rita
	Massachusetts General Hospital	Bardia, Aditya
	MGH Newton-Wellesley Hospital's Vernon Cancer Center	Bardia, Aditya

	University of Texas-MD Anderson Cancer Center	Valero, Vicente
	Mayo Clinic	Northfelt, Donald
	Meritus Center for Clinical Research	McCormack, Michael
	Meridian Health	Waintraub, Stanley
	Tennessee Oncology, PLLC/The Sarah Cannon Research Institute	Yardley, Denise
	Northwest Cancer Specialists, P.C.	Andersen, Jay
	Virginia Oncology Associates	Danso, Michael
	Texas Oncology-Dallas Presbyterian Hospital	McIntyre, Kristi
	Southern Cancer Center, PC	Meshad, Michael
	Texas Oncology-Austin Central	Patt, Debra
	Oncology & Hematology Associates of Southwest Virginia, Inc., DBA Blue Ridge Cancer Care	Richards, Paul
	Texas Oncology-Tyler	Davis, Sasha
	Maryland Oncology Hematology, P.A.	Wallmark, John
	Arizona Oncology Associates PC- HOPE	Swart, Rachel
	Texas Oncology-Baylor Charles A. Sammons Cancer Center	OShaughnessy, Joyce
	Texas Oncology- Plano East	Stokoe, Christopher
	Virginia Cancer Specialists, PC	Favret, Anne
	Texas Oncology-Memorial City	Cairo, Michelina
	Rocky Mountain Cancer Centers LLP	Robinson, Sara
Ukraine	MI Kryviy Rih Center of Dnipropetrovsk Regional Council	Adamchuk, Hryhoriy
	MI Odesa Regional Clinical Hospital	Berzoy, Oleksandr
	Dnipropetrovsk City Multidiscipline Clinical Hosp. 4 of DRC	Bondarenko, Igor
	Kyiv City Clinical Hospital 2	Cheshuk, Valeriy
	Khmelnitskiy Regional Onkology Dispensary	Drobner, Igor
	Zaporozhye Regional Clinical Hospital	Kolesnik, Oleksii
	Zaporizhzhya Regional Clinical Oncology Center	Kovalyov, Olexiy
	MI Precarpathian Clinical Oncology Center	Kryzhanivska, Anna
	Grigoriev Institute for medical Radiology NAMS of Ukraine	Nasonova, Alla/Popovska, Tetiana
	Regional Clinical Specialized Dispensary of Pop. Radiation Protection	Neffa, Maryna
	Communal non profit enterprise Regional Clinical Oncology Center	Kucheryava, Nadya/Otchenash, Natalya
	Vinnitsya Regional Clinical Oncological Dispensary	Shevnya, Sergii
	Communal nonprofit enterprise "Kherson Regional Oncology Dispensary" of Kherson Regional Council	Sokur, Iryna
	MI Odessa Regional Oncological Centre	Krasnohrud, Yuliia
	PP PPC Acinus Medical and Diagnostic Centre	Ursol, Grygorii

	Kyiv City Clinical Oncology Centre	Voitko, Nataliia/Osinsky, Dmytro
	RMI - Sumy Regional Clinical Oncology Dispensary	Vynnychenko, Ihor
	National Cancer Institute of the MoH of Ukraine	Ostapenko, Yuriy
	Medical center of the Limited Liability Company Yulis	Kovalyov, Olexiy
	Zhytomyr Regional Oncology Center	Lipetska, Oksana

Steering Committee

Name	Institution	Country
Fabrice Andre	Institut Gustave Roussy	France
Aditya Bardia (co-chair)	Massachusetts General Hospital<br>MGH - North Shore Cancer Center<br>MGH Newton-Wellesley Hospital's Vernon Cancer Center*	United States
Fatima Cardoso (chair)	Champalimaud Clinical Center/Champalimaud Foundation	Portugal
David Cescon	Princess Margaret Cancer Centre	Canada
Javier Cortes Castan	Hospital Beata María Ana-oncology	Spain
Nadia Harbeck	Klinikum der Universitaet Muenchen - Grosshadern	Germany
Heather McArthur	Cedars Sinai Medical Center Samuel Oschin Comp. Cancer Institute	United States
Peter Schmid	Barts Health NHS Trust	United Kingdom
Melinda Telli	Stanford University School of Medicine/Standard Cancer Institute	United States

*At the time of patient enrollment in study

Data Monitoring Committee

Name	Affiliation	Country
Massimo Cristofanilli, MD<br>(CHAIR)	Weill Cornell Medicine/New York-Presbyterian Hospital	United States
Kathy Miller, MD	Professor of Medicine, Breast Cancer Program Co-Leader, Indiana University School of Medicine	United States
James J. Dignam, PhD	University of Chicago, Department of Public Health Sciences, The University of Chicago	United States
Kevin Kalinsky	Associate Professor, Department of Hematology and Medical Oncology, Emory University School of Medicine	United States

Eligibility Criteria

Inclusion Criteria

An individual is eligible for inclusion in the study if the individual meets all of the following criteria:

Type of Participant and Disease Characteristics

1. Participant has a localized invasive breast ductal adenocarcinoma, confirmed by the local pathologist, that includes either T1c-T2 (tumor size ≥2 cm), clinical node stage (cN)1-cN2, or T3-T4, cN0-cN2.

   Note: Multifocal tumors defined as the presence of 2 or more foci of cancer within the same quadrant are allowed; at least 1 of the tumors needs to be ≥2 cm. ER+/HER2– status needs to be confirmed for each focus.

   Note: Inflammatory breast cancer is allowed.

   Note: Participants with node negative disease will be capped at 20% of the total population.

2. Has centrally confirmed ER+/HER2–, Grade 3 breast cancer of ductal histology, according to the most recent American Society of Clinical Oncology/College of American Pathologist guidelines.
3. Provides a new or recently obtained core needle biopsy, consisting of multiple cores, taken from the primary breast tumor(s) for central determination of HR status (ER and progesterone receptor), HER2, grade, and PD-L1 status.

   Note: Adequacy of the biopsy specimen for the above analyses must be confirmed by the central laboratory. Submission of another tumor specimen may be required, if adequate tumor tissue was not provided the first time.

Note: Sponsor agreement is required for formalin-fixed paraffin-embedded (FFPE) tumor tissue sample or slides that were obtained greater than 60 days prior to the date that the documented informed consent was obtained.

Demographics

4. Is a male or female ≥18 years of age on the day of signing informed consent.
5. Has an Eastern Cooperative Oncology Group (ECOG) performance status of 0 or 1, as assessed within 10 days prior to initiation of study treatment.

Male Participants

6. A male participant must agree to use a contraception during the treatment period and for at least 12 months (for participants who received cyclophosphamide) or 6 months (for participants who did not receive cyclophosphamide) after the last dose of study treatment and refrain from donating sperm during this period.

Female Participants

7. A female participant is eligible to participate if she is not pregnant, not breastfeeding, and at least one of the following conditions applies:
   a. Not a woman of childbearing potential (WOCBP).

   OR

   b. A WOCBP who agrees to follow the contraceptive guidance during the treatment period and for at least 12 months (for participants who received cyclophosphamide) or 6 months (for participants who did not receive cyclophosphamide) after the last dose of study treatment with pembrolizumab or placebo.

Informed Consent

8. The participant (or legally acceptable representative if applicable) provides documented informed consent for the study. The participant may also provide consent for future biomedical research. However, the participant may participate in the main study without participating in future biomedical research.

Laboratory Evaluations

9. Has adequate organ function as detailed in the **Table**; all screening laboratory tests should be performed within 10 days prior to initiation of study treatment.

Table. Adequate Organ Function Laboratory Values

**System**	**Laboratory Value**
**Hematological**	
Absolute neutrophil count	≥1,500 cells/µL
Platelets[a]	≥100,000 cells/µL
Hemoglobin[a]	≥9 g/dL or ≥5.6 mmol/L
**Renal**	
Creatinine **<u>OR</u>**<br>Measured or calculated[b] creatinine clearance<br>(GFR can also be used instead of CrCl)	≤1.5 × ULN **<u>OR</u>**<br>≥50 mL/min for participants with creatinine levels<br>≥1.5 × institutional ULN
**Hepatic**	
Total bilirubin	≤1.5 × ULN **<u>OR</u>**<br>Direct bilirubin ≤ULN for participants with total bilirubin levels ≥1.5 × ULN
AST (SGOT) and ALT (SGPT)	≤2.5 × ULN
**Coagulation**	
INR or PT<br>Activated partial thromboplastin time (aPTT)<br>or partial thromboplastin time (PTT)	≤1.5 × ULN unless participant is receiving anticoagulant therapy as long as PT or aPTT/PTT is within therapeutic range of intended use of anticoagulants

ALT, alanine aminotransferase; AST, aspartate aminotransferase; CrCl, creatinine clearance; GFR, glomerular filtration rate; INR, international normalized ratio; PT, prothrombin time; SGOT, serum glutamic oxaloacetic transaminase; SGPT, serum glutamic pyruvic transaminase; ULN, upper limit of normal.

[a]Platelet and hemoglobin requirements cannot be met by use of recent transfusion or growth factor support (granulocyte colony-stimulating factor [G-CSF], granulocyte-macrophage colony-stimulating factor [GM-CSF], or erythropoietin) within 2 weeks prior to initiation of study treatment.

[b]Creatinine clearance should be calculated per institutional standard.

Exclusion Criteria

An individual must be excluded from the study if the individual meets any of the following criteria:

Medical Conditions

1. Has a history of non-infectious pneumonitis that required treatment with steroids or has current pneumonitis.
2. Has breast cancer with lobular histology.
3. Has bilateral invasive breast cancer.
4. Has metastatic (Stage IV) breast cancer.
5. Has multi-centric breast cancer (presence of more than 1 tumor in different quadrants of the breast).
6. Has any of the following clinical lymph node staging per current AJCC staging criteria for breast cancer staging based on radiological and/or clinical assessment: cN3, cN3a, cN3b, or cN3c.
7. Has ER–, progesterone receptor-positive breast cancer.
8. Participants who have undergone excisional biopsy of the primary tumor and/or axillary lymph nodes or have undergone sentinel lymph node biopsy prior to study treatment.
9. Has a known additional, invasive, malignancy that is progressing or required active treatment in the last 5 years.

   Note: Participants with basal cell carcinoma of the skin, squamous cell carcinoma of the skin, breast ductal carcinoma in situ, or cervical carcinoma in situ that has undergone potentially curative therapy are not excluded.

10. Has a diagnosis of immunodeficiency or is receiving chronic systemic steroid therapy (dosing exceeding 10 mg daily of prednisone equivalent) or any other form of immunosuppressive therapy within 7 days prior to the first dose of study treatment.
11. Has an active autoimmune disease that has required systemic treatment in the past 2 years (i.e., with use of disease modifying agents, corticosteroids, or immunosuppressive drugs).

    Note: Replacement therapy (e.g., thyroxine, insulin, or physiologic corticosteroid replacement therapy for adrenal or pituitary insufficiency) is not considered a form of systemic treatment.

12. Has a known history of active tuberculosis (Bacillus tuberculosis).
13. Has an active infection requiring systemic therapy.
14. Has a history or current evidence of any condition (e.g., transfusion-dependent anemia or thrombocytopenia), therapy, or laboratory abnormality that is specifically contraindicated per the current locally-approved labeling, that might confound the results of the study, interfere with the participant's involvement for the full duration of the study, or is not in the best interest of the participant to be involved, in the opinion of the treating investigator.
15. Has known psychiatric or substance abuse disorders that would interfere with cooperation with the requirements of the study.
16. Has left ventricular ejection fraction (LVEF) of <50% or below the institution limit of normal, as assessed by echocardiogram (ECHO) or multigated acquisition (MUGA) scan performed at screening.
17. Has other significant cardiac disease, such as:
    a. History of myocardial infarction, acute coronary syndrome, or coronary angioplasty/stenting/bypass within the last 6 months;

b. Congestive heart failure (CHF) New York Heart Association (NYHA) Class II-IV or history of CHF NYHA Class III or IV.

18. Has a known history of human immunodeficiency virus (HIV) infection.

    Note: No HIV testing is required unless mandated by local health authority.

19. Has a known history of hepatitis B (defined as hepatitis B surface antigen [HbsAg] reactive) or known active hepatitis C virus (defined as HCV RNA [qualitative] is detected) infection.

    Note: No testing for hepatitis B or hepatitis C is required unless mandated by local health authority.

20. A WOCBP who has a positive urine pregnancy test within 72 hours before the first dose of study treatment. If the urine test cannot be confirmed as negative, a serum pregnancy test is required. In such cases, the participant must be excluded from participation if the serum pregnancy result is positive.

Prior/Concomitant Therapy

21. Has received prior treatment for breast cancer.
22. Has received prior therapy with an anti-PD-1, anti-PD-L1, or anti-PD-L2 agent or with an agent directed to another stimulatory or co-inhibitory T-cell receptor (e.g., CTLA-4, OX 40, CD137).
23. Has received a live vaccine within 30 days prior to the first dose of study treatment. Examples of live vaccines include, but are not limited to, the following: measles, mumps, rubella, varicella/zoster (chicken pox), yellow fever, rabies, Bacillus Calmette–Guérin (BCG), typhoid, and intranasal influenza vaccines (eg, FluMist®) vaccine.

Note: Seasonal influenza vaccines for injection are generally killed virus vaccines and are allowed.

24. Has severe hypersensitivity (≥Grade 3) to any of the components or excipients used in the study treatments.

Prior/Concurrent Clinical Study Experience

25. Is/was enrolled in a study of an investigational agent and received study therapy, or used an investigational device within 4 weeks (12 months for an investigational agent or device with anticancer or antiproliferative properties) prior to the first dose of study treatment.

    Note: Participants who have entered the follow-up phase of an investigational study may participate as long as 4 weeks (12 months, for an investigational agent or device with anticancer or antiproliferative properties) have elapsed since the last dose of the previous investigational agent or last use of investigational device.

Other Exclusions

26. Is pregnant, breastfeeding, or expecting to conceive or father children within the projected duration of the study, starting with the screening visit through 12 months (for participants who received cyclophosphamide) or 6 months (for participants who did not receive cyclophosphamide) after the last dose of study treatment.
