# Supplementary material for: Pembrolizumab and chemotherapy in high-risk, early-stage, ER+/HER2− breast cancer: a randomized phase 3 trial
Source: Nat Med. 2025 Jan 21;31(2):442–8. doi: 10.1038/s41591-024-03415-7 (PMC11835712; doi:10.1038/s41591-024-03415-7)
Supplement: Supplementary file 2 — Reporting Summary [file 41591_2024_3415_MOESM2_ESM.pdf]

nature portfolio

Corresponding author(s): Fatima Cardoso

Last updated by author(s): Oct 24, 2024

Reporting Summary

Nature Portfolio wishes to improve the reproducibility of the work that we publish. This form provides structure for consistency and transparency in reporting. For further information on Nature Portfolio policies, see our Editorial Policies and the Editorial Policy Checklist.

Statistics

For all statistical analyses, confirm that the following items are present in the figure legend, table legend, main text, or Methods section.

n/a	Confirmed	
☐	☒	The exact sample size (n) for each experimental group/condition, given as a discrete number and unit of measurement
☒	☐	A statement on whether measurements were taken from distinct samples or whether the same sample was measured repeatedly
☐	☒	The statistical test(s) used AND whether they are one- or two-sided<br>*Only common tests should be described solely by name; describe more complex techniques in the Methods section.*
☐	☒	A description of all covariates tested
☐	☒	A description of any assumptions or corrections, such as tests of normality and adjustment for multiple comparisons
☐	☒	A full description of the statistical parameters including central tendency (e.g. means) or other basic estimates (e.g. regression coefficient) AND variation (e.g. standard deviation) or associated estimates of uncertainty (e.g. confidence intervals)
☐	☒	For null hypothesis testing, the test statistic (e.g. F, t, r) with confidence intervals, effect sizes, degrees of freedom and P value noted<br>*Give P values as exact values whenever suitable.*
☒	☐	For Bayesian analysis, information on the choice of priors and Markov chain Monte Carlo settings
☒	☐	For hierarchical and complex designs, identification of the appropriate level for tests and full reporting of outcomes
☐	☒	Estimates of effect sizes (e.g. Cohen's d, Pearson's r), indicating how they were calculated

Our web collection on statistics for biologists contains articles on many of the points above.

Software and code

Policy information about availability of computer code

Data collection	Clinical data were captured in the study clinical database.
Data analysis	Statistical analyses were conducted using SAS version 9.4 (SAS Institute, Cary, NC).

For manuscripts utilizing custom algorithms or software that are central to the research but not yet described in published literature, software must be made available to editors and reviewers. We strongly encourage code deposition in a community repository (e.g. GitHub). See the Nature Portfolio guidelines for submitting code & software for further information.

Data

Policy information about availability of data

All manuscripts must include a data availability statement. This statement should provide the following information, where applicable:

- Accession codes, unique identifiers, or web links for publicly available datasets
- A description of any restrictions on data availability
- For clinical datasets or third party data, please ensure that the statement adheres to our policy

Merck Sharp & Dohme LLC, a subsidiary of Merck & Co., Inc., Rahway, NJ, USA (MSD) is committed to providing qualified scientific researchers access to anonymized data and clinical study reports from the company's clinical trials for the purpose of conducting legitimate scientific research. MSD is also obligated to protect the rights and privacy of trial participants and, as such, has a procedure in place for evaluating and fulfilling requests for sharing company clinical trial data with qualified external scientific researchers. The MSD data sharing website (available at: http://engagezone.msd.com/ds_documentation.php) outlines the process and

requirements for submitting a data request. Applications will be promptly assessed for completeness and policy compliance. Feasible requests will be reviewed by a committee of MSD subject matter experts to assess the scientific validity of the request and the qualifications of the requestors. In line with data privacy legislation, submitters of approved requests must enter into a standard data-sharing agreement with MSD before data access is granted. Data will be made available for request after product approval in the US and EU or after product development is discontinued. There are circumstances that may prevent MSD from sharing requested data, including country- or region-specific regulations. If the request is declined, it will be communicated to the investigator. Access to genetic or exploratory biomarker data requires a detailed, hypothesis-driven statistical analysis plan that is collaboratively developed by the requestor and MSD subject matter experts; after approval of the statistical analysis plan and execution of a data-sharing agreement, MSD will either perform the proposed analyses and share the results with the requestor or will construct biomarker covariates and add them to a file with clinical data that is uploaded to an analysis portal so that the requestor can perform the proposed analyses.

Research involving human participants, their data, or biological material

Policy information about studies with human participants or human data. See also policy information about sex, gender (identity/presentation), and sexual orientation and race, ethnicity and racism.

Reporting on sex and gender	The manuscript reports on patients with breast cancer.
Reporting on race, ethnicity, or other socially relevant groupings	Patients were stratified at randomization by region (Eastern Europe vs China vs all other countries). Relevant baseline characteristics are reported in Table 1.
Population characteristics	This information is provided in Table 1.
Recruitment	Patients were enrolled from 222 global sites. Recruitment was done by investigators and was limited to patients who met protocol-specified criteria for eligibility. Details of these sites are provided in the Supplemental Material.
Ethics oversight	The protocol was approved by an ethics body at each institution. Patients provided written informed consent. An external independent data monitoring committee oversaw the study, periodically assessed safety, and assessed efficacy at prespecified interim analyses (see Supplementary Material for details).

Note that full information on the approval of the study protocol must also be provided in the manuscript.

Field-specific reporting

Please select the one below that is the best fit for your research. If you are not sure, read the appropriate sections before making your selection.

☒ Life sciences ☐ Behavioural & social sciences ☐ Ecological, evolutionary & environmental sciences

For a reference copy of the document with all sections, see nature.com/documents/nr-reporting-summary-flat.pdf

Life sciences study design

All studies must disclose on these points even when the disclosure is negative.

Sample size	With enrollment of approximately 1240 patients, the trial had >99% power to detect a true difference of 15 percentage points for the comparison of the rate of pCR (stage ypT0/Tis ypN0) between the treatment arms, at a one-sided alpha level of 0.005. It would have a power of 84% to detect a hazard ratio for EFS of 0.73, at a one-sided alpha level of 0.02 at the final analysis. The full statistical analysis plan is provided in the protocol.
Data exclusions	Efficacy was evaluated in the intention-to-treat population, which included all patients who had undergone randomization. Safety was evaluated in the as-treated population, which included all patients who had undergone randomization and received ≥1 trial drug, underwent surgery, or both.
Replication	This was a clinical trial. Replication was not appropriate.
Randomization	Patients were randomly assigned (in a 1:1 ratio) to the pembrolizumab–chemotherapy arm or the placebo–chemotherapy arm using a central interactive voice-response system with an integrated Web-response system.
Blinding	This was a double-blind study.

Reporting for specific materials, systems and methods

We require information from authors about some types of materials, experimental systems and methods used in many studies. Here, indicate whether each material, system or method listed is relevant to your study. If you are not sure if a list item applies to your research, read the appropriate section before selecting a response.

Materials & experimental systems

n/a	Involved in the study	
☒	☐	Antibodies
☒	☐	Eukaryotic cell lines
☒	☐	Palaeontology and archaeology
☒	☐	Animals and other organisms
☐	☒	Clinical data
☒	☐	Dual use research of concern
☒	☐	Plants

Methods

n/a	Involved in the study	
☒	☐	ChIP-seq
☒	☐	Flow cytometry
☒	☐	MRI-based neuroimaging

Clinical data

Policy information about clinical studies

All manuscripts should comply with the ICMJE guidelines for publication of clinical research and a completed CONSORT checklist must be included with all submissions.

Clinical trial registration	ClinicalTrials.gov, NCT03725059
Study protocol	The study protocol is available upon request (per data availability statement)
Data collection	From December 27, 2018 through August 5, 2022, a total of 1278 patients from 222 global sites were enrolled in the study.
Outcomes	The study's primary endpoints were pCR, defined as ypT0/Tis ypN0 at the time of surgery, and EFS in the intention-to-treat population. Secondary endpoints included pCR according to the definitions of ypT0 ypN0 and ypT0/Tis in all patients, pCR according to all definitions in patients with PD-L1 CPS ≥1 tumors, EFS among patients with PD-L1 CPS ≥1 tumors, and OS among all patients and patients with PD-L1 CPS ≥1 tumors.

Plants

Seed stocks	*Report on the source of all seed stocks or other plant material used. If applicable, state the seed stock centre and catalogue number. If plant specimens were collected from the field, describe the collection location, date and sampling procedures.*
Novel plant genotypes	*Describe the methods by which all novel plant genotypes were produced. This includes those generated by transgenic approaches, gene editing, chemical/radiation-based mutagenesis and hybridization. For transgenic lines, describe the transformation method, the number of independent lines analyzed and the generation upon which experiments were performed. For gene-edited lines, describe the editor used, the endogenous sequence targeted for editing, the targeting guide RNA sequence (if applicable) and how the editor was applied.*
Authentication	*Describe any authentication procedures for each seed stock used or novel genotype generated. Describe any experiments used to assess the effect of a mutation and, where applicable, how potential secondary effects (e.g. second site T-DNA insertions, mosiacism, off-target gene editing) were examined.*
